# Mesoporous Silica-Coated Upconverting Nanorods for Singlet Oxygen Generation: Synthesis and Performance

**DOI:** 10.3390/ma14133660

**Published:** 2021-06-30

**Authors:** Zhen Zhang, Xiao-Lian Zhang, Bin Li

**Affiliations:** 1 School of Chemistry and Chemical Engineering, Gannan Normal University, Ganzhou 341000, China; zhangzhen@163.com; 2 Changchun Institute of Optics, Fine Mechanics and Physics, University of Chinese Academy of Sciences, Changchun 130033, China; ciomplibin@163.com

**Keywords:** upconverting, photodynamic therapy, singlet oxygen generation

## Abstract

Photodynamic therapy (PDT) has been reported as a possible pathway for the treatment of tumors. The exploration for promising PDT systems thus attracts continuous research efforts. This work focused on an ordered core–shell structure encapsulated by mesoporous SiO_2_ with the upconverting emission property following a surfactant-assisted sol–gel technique. The mesoporous silica shell possessed a high surface area-to-volume ratio and uniform distribution in pore size, favoring photosensitizer (rose bengal) loading. Simultaneously, upconverting nanocrystals were synthesized and used as the core. After modification via hydrophobic silica, the hydrophobic upconverting nanocrystals became hydrophilic ones. Under near-infrared (NIR) light irradiation, the nanomaterials exhibited strong green upconverting luminescence so that rose bengal could be excited to produce singlet oxygen. The photodynamic therapy (PDT) feature was evaluated using a ^1^O_2_ fluorescent indicator. It was found that this core–shell structure generates ^1^O_2_ efficiently. The novelty of this core–shell structure was the combination of upconverting nanocrystals with a mesoporous SiO_2_ shell so that photosensitizer rose bengal could be effectively adsorbed in the SiO_2_ shell and then excited by the upconverting core.

## 1. Introduction

It has been reported that tumor tissues are sensitive to some photosensitive reagents when being exposed to an appropriate irradiation wavelength which makes photodynamic therapy (PDT) a candidate for effective and economical treatment method for body tumors and premalignant conditions [1]. It is well-known that PDT works by way of a photochemical reaction between photoexcited photosensitizers (PS) and O_2_ molecules. The excited photosensitizer molecules can interact with nearby oxygen molecules, generating singlet oxygen ^1^O_2_, causing damage to tumor cells and ultimately their death [2,3]. Regardless of the promising future of PDT in the field of cancer treatment, there are still problems to be solved. For example, the penetration depth of excitation light in a living body is still too short to be applied; some photosensitizer molecules are incompatible with water-based environments; their photostability under excitation light is unsatisfied [4,5,6,7,8]. Actually, the currently reported photosensitizer molecules for PDT application are usually driven by blue or even ultraviolet (UV) light. As a consequence, the penetration depth is unsatisfactory in a living body, which prevents PDT from clinical application. Compared to visible and UV irradiation, near-infrared (NIR) irradiation is a promising way to improve penetration depth owing to the low absorption in a living body; background light interference and photodamage to normal tissues are greatly avoided [9]. The application of RE-based upconverting nanomaterials which transform NIR radiation to higher-energy excitation light may offer a new pathway for PDT. They present good photostability, low toxicity, and unique emission bands upon NIR excitation [10,11,12,13,14,15]. Upconverting nanosystem-based PDT materials claim an appropriate photosensitizer structure with proper absorbance for upconverting emission. Upconverting luminescence can excite neighboring photosensitizer molecules to initiate the PDT effect. Till now, there have been some reports about PDT systems based on upconverting nanomaterials such as silica coatings, polymer immobilization, and hydrophobic reactions [16,17,18,19,20,21,22].

Recently, porous silica-derived nanomaterials have been attracting more and more research interest as a host for pharmaceutical delivery and fluorescent labeling in virtue of their large surface-area-to-volume ratio, homogeneous porous structure, favorable biocompatibility, and stability in living tissues [23,24,25,26,27,28,29]. Combining porous silica materials with a functional component via a hybrid structure has been reported as a promising protocol. Here, porous silica materials offer a sufficient number of surface sites for the immobilization of functional component molecules, and their porous structure accelerates analyte adsorption and accumulation for later reaction with functional component molecules [30]. Hence, photosensitizer molecules are dispersed into silica channels with a high doping level to be conveniently absorbed by living tissues [31,32,33,34]. As for the upconverting nanomaterials, the NaYF_4_ lattice is the most widely reported one owing to its high upconverting efficiency and good stability with controllable photoluminescence (PL), adjustable size, and good biocompatibility [35,36,37,38,39,40,41,42]. Zhang et al. were the first to demonstrate NaYF_4_ upconverting nanoparticles for PDT application, which sparked the following research [43].

In this work, we synthesized an ordered core–shell structure encapsulated by mesoporous silica with the upconverting emission property following a surfactant-assisted sol–gel technique. The β-NaYF_4_ nanocrystals were coated by a thin layer of silica and a mesoporous SiO_2_ layer, which still showed strong upconverting emission. At the same time, the porous silica layer had sufficiently large channels, offering immobilization sites for photosensitizer molecules (rose bengal). Here, rose bengal had strong absorption for the upconverting emission from NaYF_4_ upconverting nanoparticles. Multicolor emission shall be generated upon NIR excitation so that the photosensitizer rose bengal could be effectively adsorbed in the SiO_2_ shell and then excited by the upconverting core. It was found that this core–shell structure generates ^1^O_2_ efficiently, making it a candidate for PDT application.

## 2. Materials and Methods

### 2.1. Reagents

All the regents used for synthesis in this paper were AR (analytical reagent) grade ones. They were used as received. Y_2_O_3_, Yb_2_O_3_, and Er_2_O_3_ were purchased from Wuxi Chemical Industrial Co. Lanthanide nitrates were obtained by reacting lanthanide oxides with nitric acid. Natural evaporation resulted in bulk crystals. EtOH (99%), acetone, NH_3_∙H_2_O (28 wt%), oleic acid (85 wt%), tetraethoxysilane (TEOS), hexadecyltrimethylammonium bromide (CTAB), rose bengal and 1,3-diphenylisobenzofuran (DPBF) were purchased from Beijing Chemical Company. Triton X-100 (tert-octylphenoxypolyethoxyethanol) was bought from Guoyao Chemistry Co. (Shanghai, China), NaOH (Beijing Chemistry Co., Beijing, China) and NaF (Tianjin Chemicals Co., Tianjin, China) were used as they were. Double distilled water was used for synthesis.

### 2.2. Synthesis of NaYF_4_ Nanocrystals

Hexagonal-phased β-NaYF_4_ nanocrystals doped with Yb^3+^ and Er^3+^ ions were synthesized through a solvothermal reaction according to the literature [44]. First, 1.4 g (35.0 mmol) of NaOH, 14.2 g (45.2 mmol) of oleic acid and 20.0 g of EtOH were mixed together. Then, 20.0 mL (16 mmol) of 0.78 M NaF solution were added dropwise. After that, 4.0 mL of 0.41 M Y(NO_3_)_3_, 2.0 mL (0.4 mmol) of 0.21 M Yb(NO_3_)_3_, and 4.0 mL (0.04 mmol) of 0.011 M Er(NO_3_)_3_ were added. After 30 min of stirring, the final mixture was sealed in a Teflon bottle and heated at 200 °C for a whole day. After cooling, the solid sample was collected and washed with EtOH and water. The crude sample was placed under 75 °C for 18 h.

### 2.3. Synthesis of Upconverting Mesoporous Silica Nanocomposites

Upconverting mesoporous silica nanocomposites were synthesized using CTAB as a mesostructural template [45]. A representative operation is described as follows. First, 0.20 g of upconverting nanocrystals are mixed with 40 mL of Triton X-100. Then, this mixture is placed in an ultrasonic bath for 12 min. After that, 160 mL of deionized H_2_O are added, and the final solution is stirred for another 8 h. The solid sample is collected and washed with EtOH and water. Then, NH_3_∙H_2_O (4.0 mL), EtOH (320 mL), and H_2_O (80 mL) are mixed with TEOS (0.12 g). Under stirring, the solid sample obtained above is added and stirred for further 8 h. The solid sample is collected, washed with EtOH and water, and mixed with CTAB (0.6 g), H_2_O (160 mL), NH_3_∙H_2_O (2.0 g), and EtOH (120 mL). The final mixture is treated under ultrasonication for 45 min. Then, TEOS (0.360 g) is added. This resulting mixture is stirred for another 8 h. The solid product is collected and washed with EtOH and H_2_O. The resulting solid sample is dispersed in acetone (80 mL) and distilled for two days so that CTAB could be removed. This CTAB procedure is performed two more times. The final solid sample is collected, washed with H_2_O, and dried for later use.

### 2.4. Adsorption of Rose Bengal Molecules

Rose bengal (5 mg) was dissolved in ethanol (10 mL) and then the as-synthesized nanocrystals were added. An ultrasonic bath was used for 12 min; then, the solvent EtOH was naturally vaporized to obtain β-NaYF_4_@SiO_2_@mSiO_2_ loaded with rose bengal.

### 2.5. Characterization

XRD (X-ray diffraction) curves were obtained using a Bruker D4 X-ray diffractometer (Bruker, Baltimore, MD, USA, Cu Ka radiation). SEM (scanning electron microscope) images were obtained using a Hitachi S-4800 microscope (Hitachi, Tokyo, Japan). N_2_ adsorption/desorption experiment was conducted on a Nova l000 analyzer (Quantachrome, Boynton Beach, FL, USA) at 77 K. Before the measurements, the samples were placed in vacuum at 100 °C for 5 h. Porous parameters of all the samples, such as surface area, pore volume, and pore size distribution, were determined by BET (Brunauer–Emmett–Teller) and BJH (Barrett–Joyner–Halenda) methods. Optical measurements, such as of absorption and emission spectra, were acquired using a Shimadzu UV-3101 spectrophotometer (Shimadzu, Tokyo, Japan) and a Hitachi F-4500 fluorescence spectrophotometer (λ_ex_ = 980 nm, Hitachi, Tokyo, Japan).

### 2.6. Determination for Singlet Oxygen Production by DPBF

In a typical DPBF experiment, DPBF was dissolved in EtOH (400 μL, 1 mM). Then, β-NaYF_4_@SiO_2_@mSiO_2_ loaded with rose bengal was added (2 mg). The solution was kept in the dark and irradiated using a 980-nm laser for 40 min, and the absorbance of DPBF at 410 nm was collected every 5 min. As a control, the solution of upconverting nanorods without loading photosensitizer molecules containing an equal amount of DPBF was measured under 980-nm irradiation.

## 3. Results and Discussion

### 3.1. Microstructure and Micromorphology

**Scheme 1:**
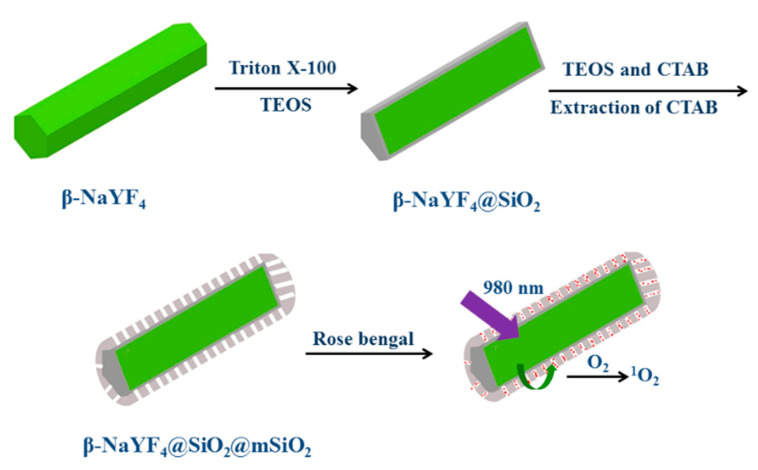
Schematic illustration of the synthesis of upconverting mesoporous silica nanocomposites.

**Figure 1:**
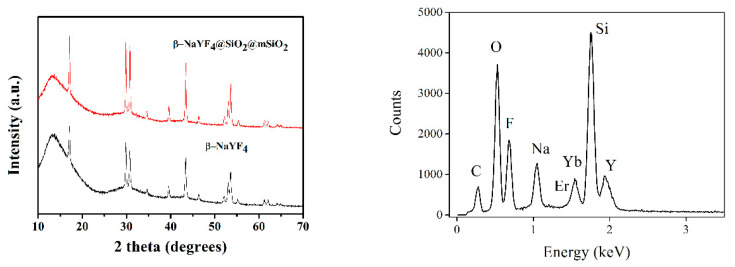
**Left**: wide-angle XRD patterns of the β-NaYF_4_ nanorods and β-NaYF_4_@SiO_2_@mSiO_2_ core–shell nanocomposites. **Right**: EDX of β-NaYF_4_@SiO_2_@mSiO_2_.

**Figure 2:**
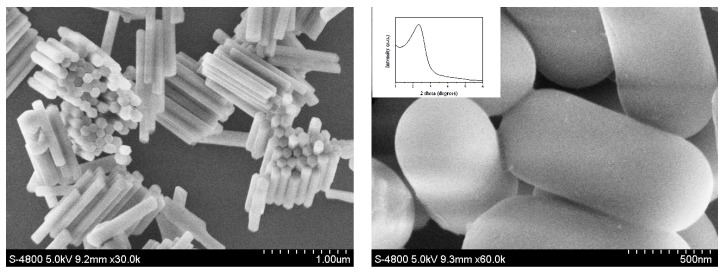
SEM images of the (**left**) β-NaYF_4_ nanorods and (**right**) β-NaYF_4_@SiO_2_@mSiO_2_ nanocomposites. Inset: Low-angle XRD pattern of the mesoporous β-NaYF_4_@SiO_2_@mSiO_2_ core–shell nanocomposites.

The synthesis protocol is presented in Scheme 1. It has been mentioned above that the upconverting mesoporous silica nanocomposites adjusted the core–shell structure and was synthesized following a surfactant-assisted sol–gel technique. Firstly, nanocrystals of β-NaYF_4_ doped with Yb(III) and Er(III) ions were synthesized and used as the core following a literature method [44]. Oleic acid was applied during synthesis to stabilize the formation of β-NaYF_4_ nanocrystals. Lanthanide nitrates and NaF served as the starting chemicals. The excess and concentrated F^–^ ions in the reaction system allowed crystal growth following the [001] direction during the hydrothermal procedure so that long rod-shaped nanocrystals were finally obtained [44]. The β-NaYF_4_ crystal nature and purity were tentatively evaluated using XRD patterns as shown in Figure 1. All the XRD peaks were found to be consistent with those of a pure hexagonal-phase β-NaYF_4_ crystal structure [44]. No sign of the mixing phase was observed. It was thus confirmed that Yb^3+^ and Er^3+^ were effectively trapped in the crystal lattice of β-NaYF_4_. As presented in the SEM image in Figure 2, the as-synthesized β-NaYF_4_ was composed of multiple long hexagonal nanorods, presenting homogeneous morphology and size. These nanorods were as wide as 120 nm in diameter and as long as 1000 nm in length. Both ends of these long nanorods exhibited hexagonal-phase morphology, indicating successful sample synthesis. These nanocrystals, however, were fully covered by a hydrophobic shell made of oleic acid (OA). Phase transfer catalyst Triton X-100 was applied here since it features both a hydrophobic end and a hydrophilic end. The former one features high affinity for oleic acid via its alkyl group. Thus, after the treatment with Triton X-100, these β-NaYF_4_ were transformed from hydrophobic to hydrophilic, which made the following Stober silica coating easy to perform. Additionally, a template reagent CTAB was introduced so that the silica-based porous shell could be established and well-grown. After the reaction, a refluxing operation extracted the CTAB template from the silica channels, leaving the pure mesoporous structure in β-NaYF_4_@SiO_2_@mSiO_2_. This statement is tentatively confirmed by the SEM image in Figure 2 (right) of β-NaYF_4_@SiO_2_@mSiO_2_. It is clear that the silica shell fully covers β-NaYF_4_ nanocrystals, presenting smooth and puffy surface. After consulting the wide-angle XRD measurement (Figure 1), it was observed that β-NaYF_4_@SiO_2_@mSiO_2_ exhibited diffraction peaks ranging from 15° to 65°, which were found to be nearly identical to the corresponding ones of β-NaYF_4_. These sharp peaks indicate good preservation of crystallinity of the β-NaYF_4_ core in β-NaYF_4_@SiO_2_@mSiO_2_, which means that the NaYF_4_ lattice was well-preserved during silica coating and modification. The EDX (energy dispersive X-ray spectroscopy) of β-NaYF_4_@SiO_2_@mSiO_2_ shown in Figure 1 (right) fully matches its desired elemental composition as shown in Figure 1 (right).

**Figure 3:**
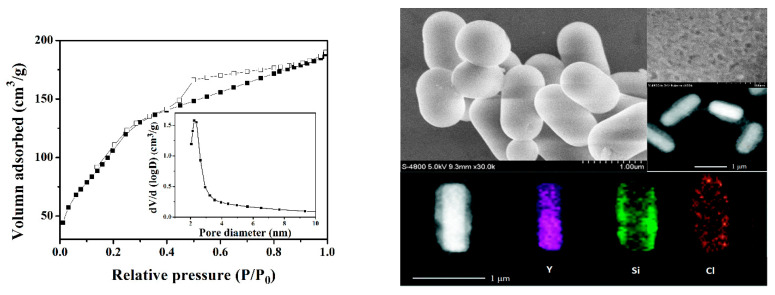
**Left**: the nitrogen adsorption−desorption isotherms and pore size distribution curve (the inset) of the mesoporous β-NaYF_4_@SiO_2_@mSiO_2_ core–shell nanocomposites. **Right**: SEM, TEM, and elemental mapping of β-NaYF_4_@SiO_2_@mSiO_2_–rose bengal.

The porous shell in β-NaYF_4_@SiO_2_@mSiO_2_ was analyzed via its low-angle XRD pattern shown in the Figure 2 inset. A strong diffraction peak of 2.3° is observed, which confirms the successful synthesis of the SiO_2_ porous shell. With the help of SEM images shown in Figure 2, it was finally confirmed that the mesoporous shell was successfully planted in β-NaYF_4_@SiO_2_@mSiO_2_. N_2_ adsorption and desorption measurements were performed on the upconverting mesoporous silica nanocomposite as shown in Figure 3. There were typical type IV isotherms according to the IUPAC (International Union of Pure and Applied Chemistry) definition for β-NaYF_4_@SiO_2_@mSiO_2_, suggesting the existence of its mesoporous shell. A narrow pore size distribution is observed in the Figure 3 inset, which shows an average pore size value of 2.30 nm. The Brunauer–Emmett–Teller (BET) surface area of β-NaYF_4_@SiO_2_@mSiO_2_ was determined to be 415 m^2^·g^−1^, while the total pore volume of β-NaYF_4_@SiO_2_@mSiO_2_ was recorded to be 0.34 cm^3^·g^−1^. These obtained nanomaterials exhibited a rather homogeneous size distribution, along with large surface area and high pore volume. These porous parameters favored photosensitizer molecule loading and consequently the improvement of the singlet oxygen generation efficiency in β-NaYF_4_@SiO_2_@mSiO_2_. Finally, the core–shell nanostructure of β-NaYF_4_@SiO_2_@mSiO_2_–rose bengal was confirmed by SEM, TEM (transmission electron microscopy), and elemental mapping images shown in Figure 3.

### 3.2. Optical Properties of Upconverting Mesoporous Silica Nanocomposite β-NaYF_4_@SiO_2_@mSiO_2_–Photosensitizer

**Figure 4:**
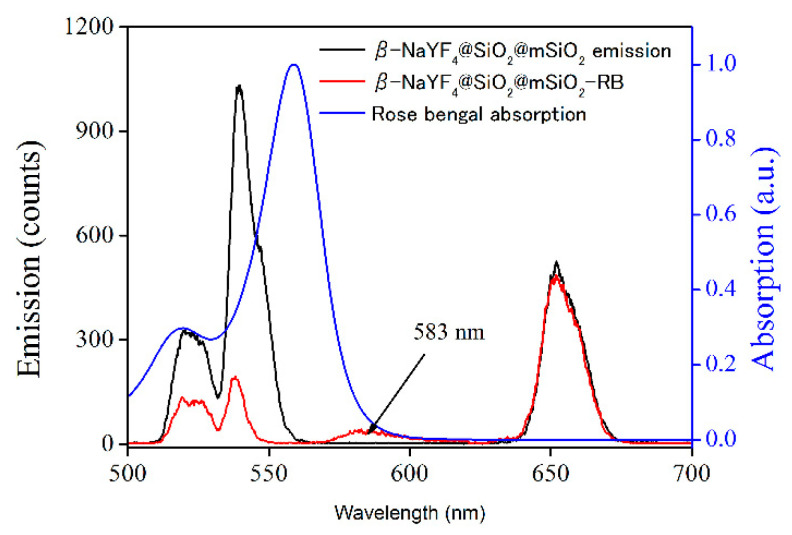
The upconverting luminescence spectra of β-NaYF_4_@SiO_2_@mSiO_2_ and β-NaYF_4_@SiO_2_@mSiO_2_–RB under 980-nm excitation and the absorption spectrum of rose bengal (red curve).

The upconverting luminescence spectrum of β-NaYF_4_@SiO_2_@mSiO_2_ upon irradiation of 980 nm and the UV-Vis absorption spectrum of photosensitizer molecules (rose bengal) can be found in Figure 4. Upon 980-nm excitation, the upconverting nanocomposite exhibited multiple upconverting emission lines, peaking at 520, 538, and 650 nm. These emission lines were generated from ^2^H_11/2_ → ^4^I_15/2_, ^4^S_3/2_ → ^4^I_15/2_, and ^4^F_9/2_ → ^4^I_15/2_ transitions of Er^3+^, respectively. It is observed in Figure 4 that the green upconverting luminescence band overlaps perfectly with the absorption spectrum of the rose bengal molecules, indicating the possibility of the rose bengal molecules absorbing green emission from the upconverting nanocore (β-NaYF_4_). Figure 4 shows the upconverting emission of β-NaYF_4_@SiO_2_@mSiO_2_–rose bengal nanocomposite after 980-nm excitation. It was observed that the two upconverting emission bands (521 nm and 539 nm) were strongly quenched, while the low-energy emission band of 651 nm was not affected. In the meanwhile, there was an emission band centering at 583 nm, corresponding to the fluorescence from rose bengal, confirming that rose bengal can efficiently accept the upconverting emission bands from the upconverting nanocore and transfer their energy to its own emission. Moreover, rose bengal is a highly efficient photosensitizer in generating ^1^O_2_ [46]. Therefore, it is predicted that upconverting luminescence excites neighboring photosensitizers to produce singlet oxygen (^1^O_2_).

### 3.3. Performance of ^1^O_2_ Production by β-NaYF_4_@SiO_2_@mSiO_2_–Rose Bengal Nanocomposite

**Figure 5:**
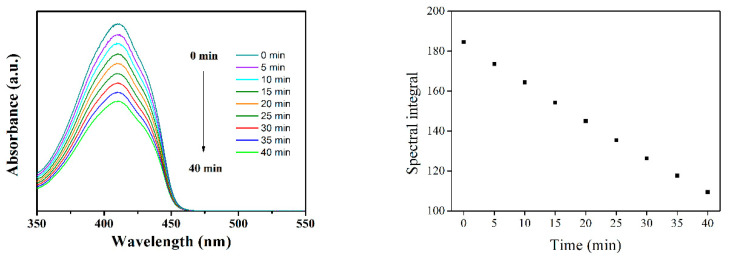
UV-Vis absorption spectra (**left**) and the corresponding spectral integral (**right**) of DPBF in the β-NaYF_4_@SiO_2_@mSiO_2_–RB solution after different irradiation times with a 980-nm laser diode.

The ^1^O_2_ production performance of β-NaYF_4_@SiO_2_@mSiO_2_-Rose-bengal nanocomposite was investigated using a reference method with 1,3-diphenylisobenzofuran as an indicator, which is a widely used singlet oxygen quencher. Singlet oxygen generation is in the inverse ratio of DPBF absorption intensity peaking at 411 nm. This is because there is an irreversible reaction between DPBF and singlet oxygen. Figure 5 shows absorption spectral monitoring for the DPBF solution upon the presence of β-NaYF_4_@SiO_2_@mSiO_2_–rose bengal when exposed to various irradiation timespans (λ_ex_ = 980 nm). The absorbance of DPBF at 410 nm displayed a continuous decrease when exposed to the 980-nm light for 40 min, which indicates successful ^1^O_2_ generation upon continuous 980-nm radiation. A linear decrease was observed for the spectral integral. In the meanwhile, the absorption peak position (410 nm) was well-preserved with no obvious spectral shift. The FWHM (full-width-at-half-maximum) values of these absorption spectra fell in the narrow region of 64.8 ± 0.4 nm. This result indicates uniform and steady generation of ^1^O_2_. This promising feature is attributed to the homogeneous distribution of the photosensitizer (rose bengal) molecules in the uniform mesopores of β-NaYF_4_@SiO_2_@mSiO_2_.

**Figure 6:**
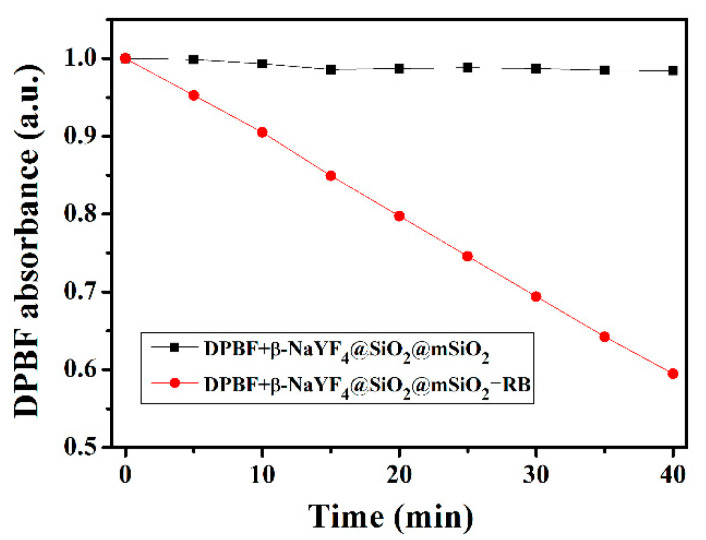
Production of singlet oxygen from β-NaYF_4_@SiO_2_@mSiO_2_–RB under 980-nm excitation in the presence of DPBF.

**Figure 7:**
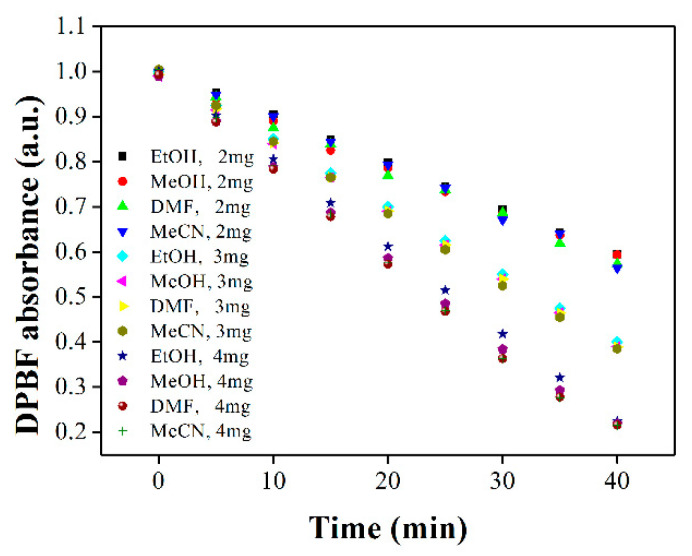
^1^O_2_ generation performance of various amounts of β-NaYF4@SiO2@mSiO2–RB in various solvents under 980-nm excitation.

In order to prove the relationship between ^1^O_2_ generation and rose bengal, a control experiment with both DPBF and upconverting nanorods but without loading photosensitizer molecules was conducted using a 980-nm laser. It is observed in Figure 6 that the DPBF absorption intensity (410 nm) was nearly unchanged. These results suggest that the rose bengal activated by emission from upconverting nanorods transports its fluorescent energy to neighboring O_2_ molecules and causes the production of singlet-state ^1^O_2_ with NIR (980 nm) excitation. Aiming at a better understanding of the ^1^O_2_ generation performance of β-NaYF_4_@SiO_2_@mSiO_2_–rose bengal, various amounts of β-NaYF_4_@SiO_2_@mSiO_2_–rose bengal were examined in various solvents, including EtOH (ethanol), MeOH (methanol), DMF (dimethyl formamidine), and MeCN (acetonitrile). It is observed in Figure 7 that, given the same amount of β-NaYF_4_@SiO_2_@mSiO_2_–rose bengal, DPBF absorption decreases in different solvents were rather similar to each other. On the other hand, with the increasing amount of β-NaYF_4_@SiO_2_@mSiO_2_–rose bengal, DPBF absorption correspondingly decreased. This result suggests that the ^1^O_2_ generation performance of β-NaYF_4_@SiO_2_@mSiO_2_–rose bengal is mainly controlled by its photosensitizer amount and has only minor correlation with the solvent used.

## 4. Conclusions

In conclusion, we demonstrated the successful synthesis of mesoporous silica coating β-NaYF_4_@SiO_2_@mSiO_2_ upconverting nanomaterials with a core–shell structure by the surfactant-assisted approach. The as-synthesized porous silica shells possessed a high surface-area-to-volume ratio and uniform distribution in pore size (BET surface area = 415 m^2^·g^−1^, pore volume = 0.34 cm^3^·g^−1^, mean pore size = 2.30 nm), favoring photosensitizer (rose bengal) loading. At the same time, the hydrophobic surface of as-synthesized NaYF_4_ nanocrystals was transformed by a covalent modification using mesoporous SiO_2_. Large pore channels were beneficial for loading photosensitizer rose bengal, the absorption spectrum of which overlapped and matched the upconverting luminescence. Under NIR light irradiation, nanomaterials exhibit strong green upconverting luminescence, which can further activate rose bengal to produce ^1^O_2_. The ^1^O_2_ generation performance of β-NaYF_4_@SiO_2_@mSiO_2_–rose bengal was evaluated using a fluorescent indicator. This result shall he helpful for future design of nanostructures and nanocomposites for potential PDT application.

## Data Availability

All data reported in this work are available upon request via corresponding author email.
